# “The Maasai Need Cows and the Cows Need Maasai,” the Use of a Photovoice Approach to Assess Animal Health Needs

**DOI:** 10.3389/fvets.2015.00046

**Published:** 2015-10-19

**Authors:** Frank van der Meer, Eoin Clancy, Adam Thomas, Susan Kutz, Jennifer Hatfield, Karin Orsel

**Affiliations:** ^1^Department of Ecosystem and Public Health, Faculty of Veterinary Medicine, University of Calgary, Calgary, AB, Canada; ^2^Department of Community Health Sciences, Cummings School of Medicine, University of Calgary, Calgary, AB, Canada; ^3^Department of Production Animal Health, Faculty of Veterinary Medicine, University of Calgary, Calgary, AB, Canada

**Keywords:** photovoice, animal health, pastoralist, photography, research methodology, Tanzania, livestock

## Abstract

The Maasai pastoralists in sub-Saharan Africa depend on their livestock for income and food. Livestock production can be significantly improved by addressing animal health concerns. We explored the use of photovoice, a participatory action research method, to strengthen our understanding of the Maasai’s animal health needs. Nine interviewees, representing warriors, elders, and women, identified animal, social, and human health themes. The use of photography provided a new medium for Maasai to express their needs and a focus for researcher–participant communications, thereby facilitating new insights across language and cultural barriers.

## Introduction

The Ngorongoro Conservation Area (NCA) in Tanzania is the homeland of pastoralists and hunter–gatherers. The predominant cultural groups in the NCA are the Maasai. Maasai are pastoralistic livestock keepers who depend on their cattle, goat, and sheep as sources of income and food (1). They are faced with a growing human population within the NCA (2), while at the same time, the cattle numbers are stable (3–6). Pastoral livestock production can be significantly improved by reducing losses through addressing the veterinary needs of livestock owners. With workshops, questionnaires, and interviews, attempts were made to identify these veterinary needs and perspectives of the Maasai herdsman (7). As a result, several programs were initiated of which the ERETO project (8) was the most comprehensive.

As a component of a joint medicine-veterinary medicine One Health field school, we initiated an animal health needs assessment. We chose to use photovoice, as this research method has been used in a variety of public health programs (9, 10). It aims at giving voice to the disadvantaged, and documenting societal realities. In an extensive review (11), it was shown that among participatory projects photovoice contributes to an enhanced understanding of community assets and needs, and this method leads to empowerment and strong motivation of the participants. While the collaborative, capacity building intent of the photovoice research approach has been clearly established, the method continues to evolve, from its inception in which narratives were blended with analog photography followed by a larger group discussion (9, 10, 12, 13) to the current use of a digital camera and a laptop followed by an interview, during which is reflected on the pictures taken. The objective of this assessment was to use photovoice to (i) build relationships with the NCA community and (ii) strengthen our understanding of their animal health needs.

## Photovoice as a Tool to Investigate Animal Health Needs

**Figure 1:**
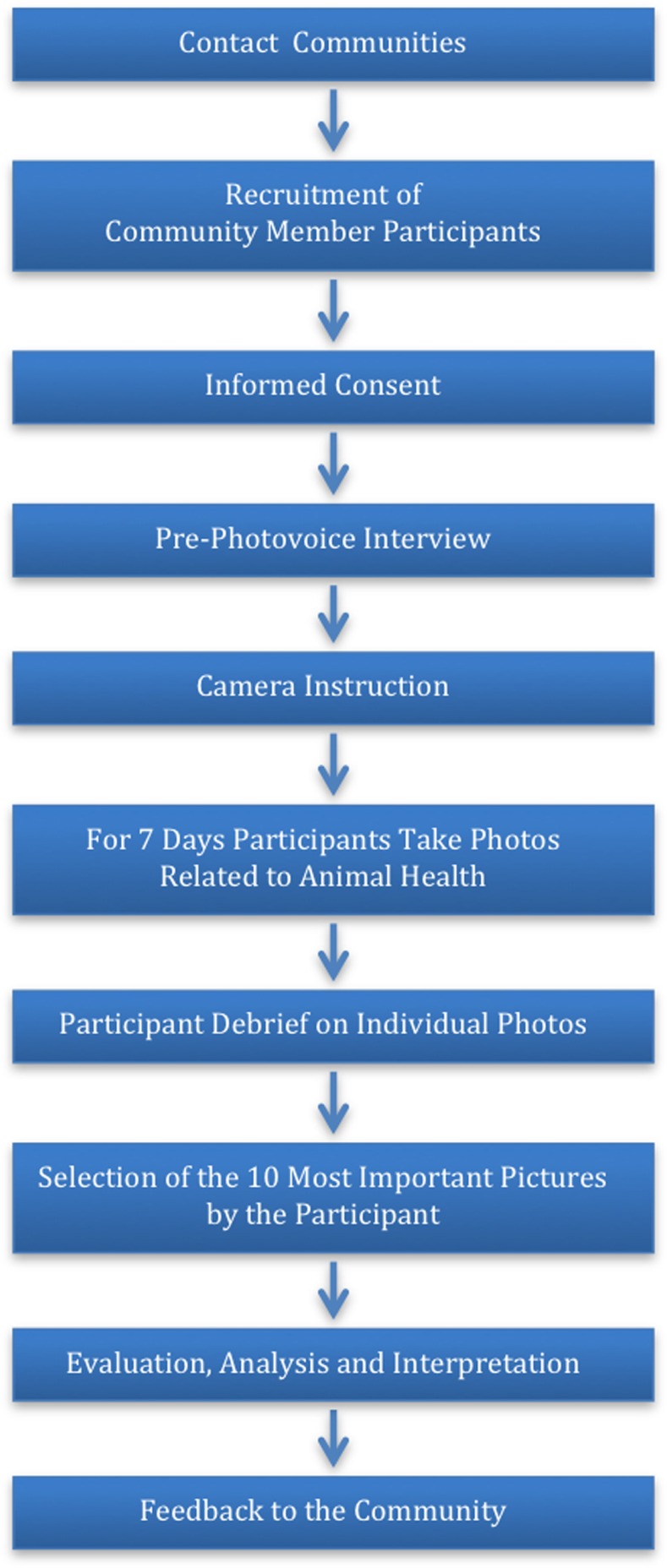
**Flow-chart of the photovoice process as used in the NCA**.

The project took place in three different ecosystems in the NCA: highland plains, semi-arid savanna, and savanna woodlands/forests (3, 4). The flow of the project is visualized in Figure 1. The photovoice method consists of several phases: (i) identification of participants, (ii) pre-interview and training in the use of cameras, photography technique and asking consent of subject, (iii) photography period, (iv) larger or smaller group discussions, and (v) data analysis. In each region, the community identified a woman, a murran (warrior, 15–35 years old), and an elder, representing the gender and age groups, to participate in the assessment. In total, nine people were interviewed. A local translator of the murran age group facilitated all interviews. The Calgary Conjoint Health Research Ethics Board and the Tanzanian National Institute for Medical Research granted ethical approval. Participants gave informed consent for the pre-photovoice interview and debrief separately. They were informed that the photos and the stories accompanying them would be used to guide research within the NCA.

An initial interview in the traditional format was performed to understand the perspectives of Maasai on animal health and their relation to animals as well as to introduce the topics for the photography part of the project. All participants were asked the following questions:

1. What are animals to the Maasai, and why are they important?
2. How are animals important for human health?
3. What is important to keep animals healthy?

## Methods

The photovoice methodology was adjusted to the NCA setting; participants used digital cameras, and the photo display and group discussion sessions were replaced with individual interviews using a laptop to display all the pictures taken by the participant for discussion. All interviews were recorded using a voice recorder. The total costs of the equipment did not exceed CDN $500. This approach allowed us to gather perspectives across the large geographic area of the NCA and acknowledge different priorities in ecosystems as well as positions in society. Equipped with an automatic digital camera, participants were directed to take pictures that best represented animal health over the next 7 days. The number of photos taken during 7 days ranged from 28 to 597. All photos were discussed, and only one participant was asked to identify the 40 most important photos. The limited photography experience of participants did not hamper the process, as only a small percentage of the pictures were unidentifiable. After having discussed all the photos in a debrief, participants were requested to select the 10 pictures that best represented their story. In the final step, the participants were invited to reflect on the photovoice process and make a comparison with the regular interview (pre-photovoice). Each recording of the debrief sessions then was transcribed and read by two researchers (Frank van der Meer and Karin Orsel). Key words were identified and clustered in three major themes: animals, human health, and social topics. The answers to the interview questions were compared to the outcomes of the post-photovoice debrief (Table 1).

**Table 1:** **Topics discussed about animal and human health needs during the pre-photovoice interview and during the photo-debrief sessions**.

Themes pre-photovoiceinterview	Topic
Animal	Feed, health, and species
	Water availability
	Use of dogs, donkey, goat, sheep, and/or bees
	Animal diseases
	Vaccination of cattle
	Treatment and prevention of animal diseases
	Antiparasitic livestock dip facilities
Social topics	Politics, economy, and education
	Orpul and other slaughter practices
Human food and health	Availability of water for human consumption
	Human health

**Themes post-photovoicedebrief**	**Topic**

Animal	Bovine breeds, feed, health, husbandry, and/or reproduction
	Water availability and/or preservation for animals
	Wildlife as threat to humans and/or animals
	Use of dogs, donkey, goat, sheep, and/or bees
	Animal diseases
	Vaccination of cattle
	Treatment and prevention of animal diseases
	Antiparasitic livestock dip facilities
Social topics	Lifestyle of Maasai
	Politics, economy, and education
	Social organization, culture, believes, and gender inequity
	Orpul and other slaughter practices
Human food and health	Availability of water for human consumption
	Human food security and food hygiene
	Human health
	Housing

## Results

Discussions arising from the pre-photovoice interview questions covered a broad range of topics directly or indirectly related to animals. Key themes and topics that emerged from the post-photovoice debrief were more extensive and detailed compared to the pre-photovoice interview. For example, the availability of feed/grass and water, the availability of safe drinking water for animals and humans, and the loss of cattle to East Coast Fever, emerged as important issues in the debrief but had not been brought up in the pre-photovoice interview. Although the participants were told that the focus of the project was animals and animal health, all participants included social and human health topics in photos taken and the subsequent post-photovoice debrief. When requested to identify the top 10 of their photos, 70% of these pictures identified animals and animal health topics.

Results from this project were brought back to the communities through community presentations. In response to the concerns about East Coast Fever, bloat and gastrointestinal parasitism, we developed and delivered workshops on the cause, signs, economics, and treatment of these diseases. In collaboration with animal health workers, and local veterinary staff visual information material was developed and distributed. The workshops were delivered in different regions of the NCA in 2013–2015, in over 15 different sessions. Approximately, 40 Maasai attended each session. A research project linking human and animal gastrointestinal parasite prevention, and treatment was also initiated in response to the photovoice discussions on worm infections and their consequences.

## Discussion and Reflections

This project was the first, information-gathering part, of a larger effort to improve animal health in the NCA. Photovoice was used to assess veterinary needs of the community as well as to build relationships with the community that should lead to an equitable partnership. As livestock are of critical importance to Maasai and their livelihoods directly depend on the health of their animals, all contacted communities were highly motivated to participate in the project.

According to the tradition, before we were born our grand parents were having cattle and they left them to our parents and now we are born and we keep cattle, so we can see that cattle are very beneficial to us as Maasai … they are very beneficial as it is medicine, food and economy …

Moran prephotovoice-interview (mod FvdM)

The 7 days given for photographs allowed them time to reflect and identify the topics that they felt were important for discussion. During the debrief, the discussion topics were entirely determined by the photographer and the detail of information that was provided was rich, providing perspective and context.

The Maasai need cows and the cows need Maasai.

Elder debrief session

Their enthusiasm led to an extensive collection of photos of variable quality. However, even low quality photos could be used for the interview as long as the participants were able to recall the reason why the photo was taken. Participants positively commented on the use of pictures as catalysts for discussion, compared to more conventional interviews, and indicated that they felt empowered by the discussion being focused around their photos. Reflecting on the photovoice method, we noticed that although confidentiality was ensured during the consent process, the major concern of the participants was that their personal opinions would be known outside of the Maasai community and potentially used for political purposes. Therefore, neither interview, debrief outcomes, nor pictures taken, were linked with names in public communications.

A translator is an integral part of the research team and the role of the translator as both an inter-cultural communicator and a data interpreter must be acknowledged in the research process (14). Gender differences can play a role in the phrasing of the answers to the interview questions and the interaction between interviewer and interviewee (15). Translators with an equal social status or gender to the interviewee would have been preferable. We used a second experienced translator with a different social status (elder) who verified the recordings and translations of the murran translator. The presence of the non-Maasai western researchers (three male and one female) might also have played a role in the way people expressed their points of view. Although the number of participants (nine) was insufficient to detect differences among regions or participants with different status in the community, the women tended to bring safe drinking water for humans forward more often, whereas the elders and murran prioritized the animals and animal health. No difference was observed in topics raised in the three different ecosystems.

The time span (7 days) for the photography period was short, but was determined due to the time constraints of the researchers. Additionally, the project occurred at the end of the rain season, therefore seasonality of animal health could not be captured.

We were surprised by the relative large number of photos that were displaying a human health topic, even though the participants were directed to focus on animal health needs. This illustrates the interrelatedness of these topics within the Maasai society and it taught our team that subsequent steps in research and interventions should use an approach that includes human and animal health in an appropriate cultural context. The key areas of concern identified with the photovoice project were used to initiate research projects.

Ok, animals are important for human health because we get milk and also we get meat from them. We sell them as well to get money and also they are important because we send children to school, we don’t have another employment, we depend on animals. So once a child passes exam and goes to school, you should sell them and then you pay school fees for them so we can see that cows are very significant.

Woman debrief session (mod FvdM)

Although the strengths of the photovoice approach are well recognized (10, 11) the disadvantages like potential risk to the participant for their expressed views, personal judgment, control of resources, as well as the challenge to analyze and summarize the content of photos should not be underestimated.

In conclusion, photovoice resulted in an expanded breadth and depth of discussions and knowledge transfer to the researchers, and participants explicitly indicated that they found it an empowering process. In addition, the outcomes directly informed us about research and outreach needs and critically re-enforced the importance of a One Health approach.

## Conflict of Interest Statement

The authors declare that the research was conducted in the absence of any commercial or financial relationships that could be construed as a potential conflict of interest; as no third party payment was received, no copyrights or patents are linked to this work or other relationships that could be perceived as potentially influencing the work.
